# Supplementary material for: Design and Deployment of a Regulation‐Compliant Infrared Heating System for UK Field Trials
Source: Plant Environ Interact. 2026 Sep 9;7(5):e70189. doi: 10.1002/pei3.70189 (PMC13555399; doi:10.1002/pei3.70189)
Supplement: Supplementary file 2 [file PEI3-7-e70189-s002.pdf]

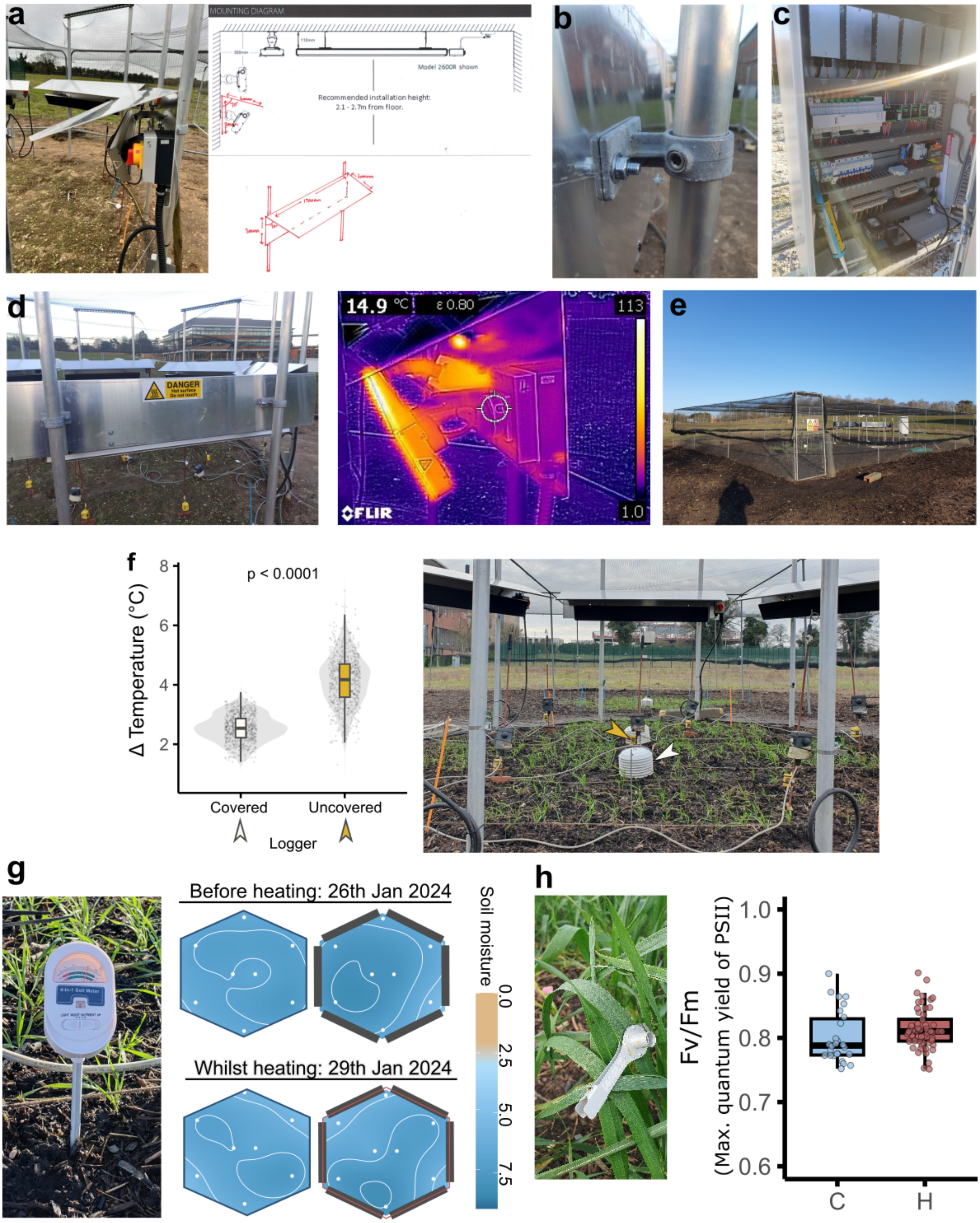
a
MOUNTING DIAGRAM
Model 2600R shown
Recommended installation height:
2.1 - 2.7m from floor.
b
c
d
DANGER
14.9 °C
ε 0.80
113
1.0
FLIR
e
f
p < 0.0001
Δ Temperature (°C)
8
6
4
2
Covered
Uncovered
Logger
g
Before heating: 26th Jan 2024
Whilst heating: 29th Jan 2024
Soil moisture
0.0
2.5
5.0
7.5
h
Fv/Fm
(Max. quantum yield of PSII)
1.0
0.9
0.8
0.7
0.6
C
H

Figure S1.

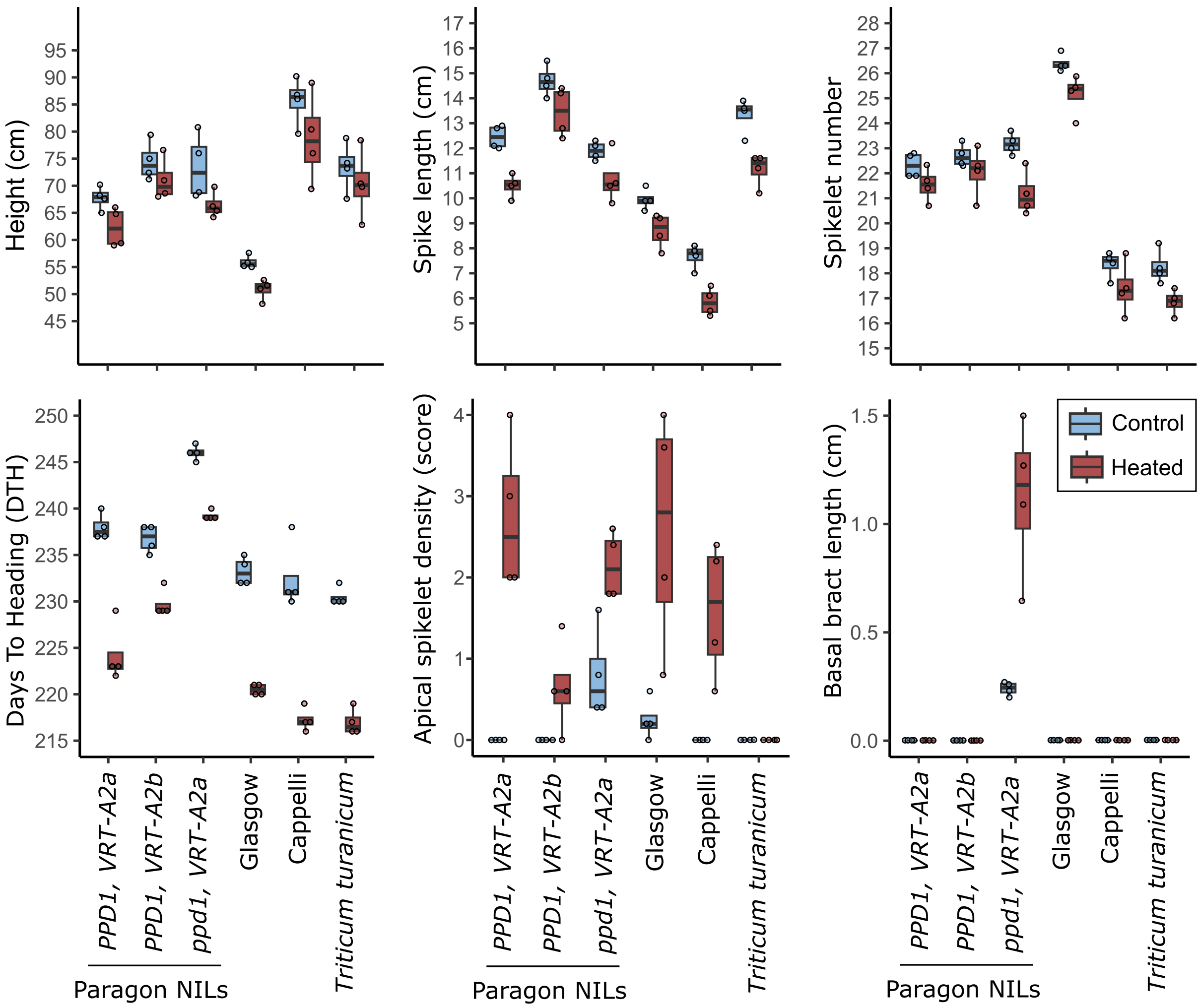

Phenotype	Treatment	Genotype	Interaction
Height	< **0.001**	< **0.001**	NS
Spike length	< **0.001**	< **0.001**	NS
Spikelet number	< **0.001**	< **0.001**	NS
DTH	< **0.001**	< **0.001**	< **0.001**
Apical spikelet density	< **0.001**	< **0.001**	< **0.001**
Basal bract length	< **0.001**	< **0.001**	< **0.001**

Figure S2.

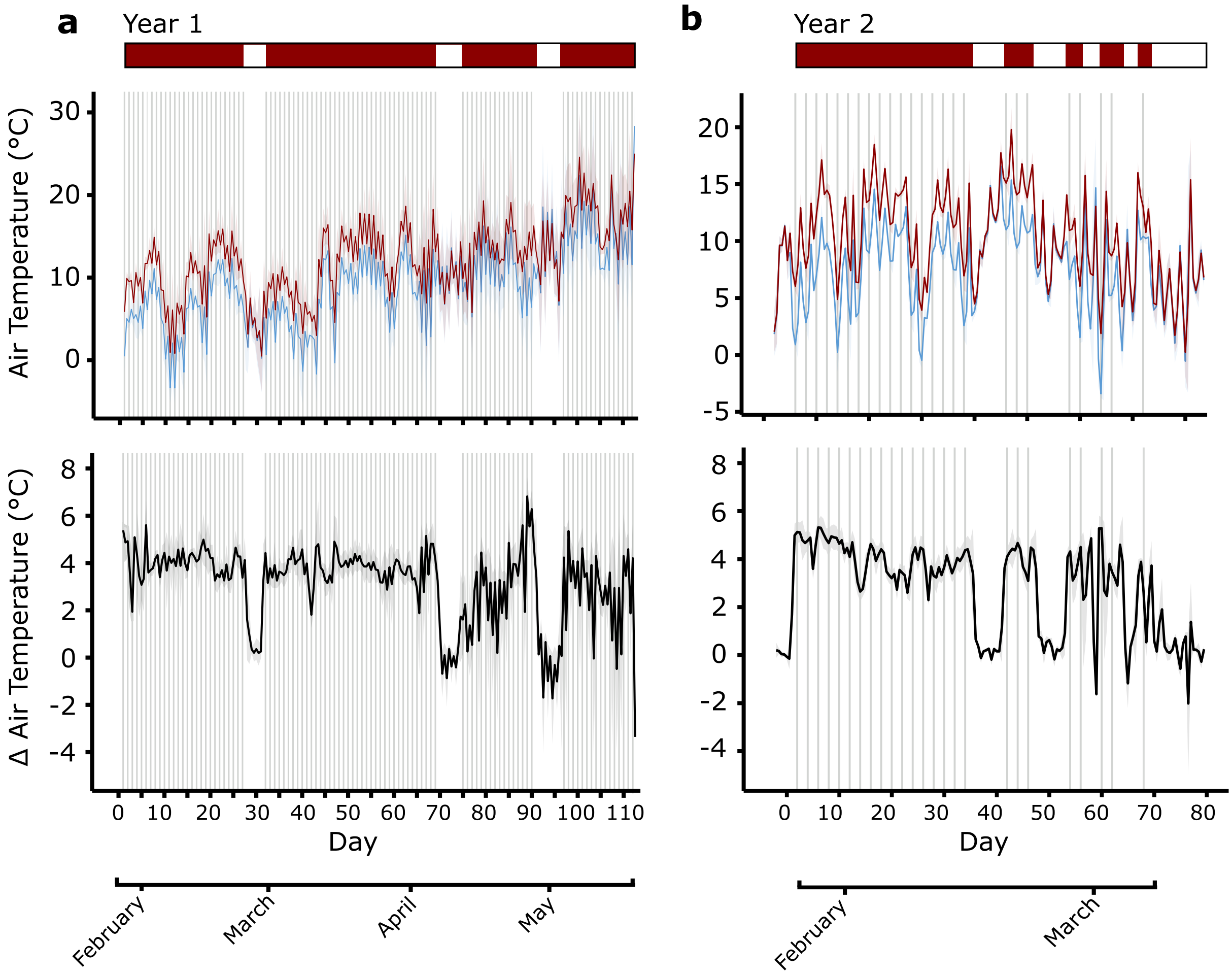
a
Year 1
b
Year 2
Air Temperature (°C)
Δ Air Temperature (°C)
Day
Day
February
March
April
May
February
March

Figure S3.

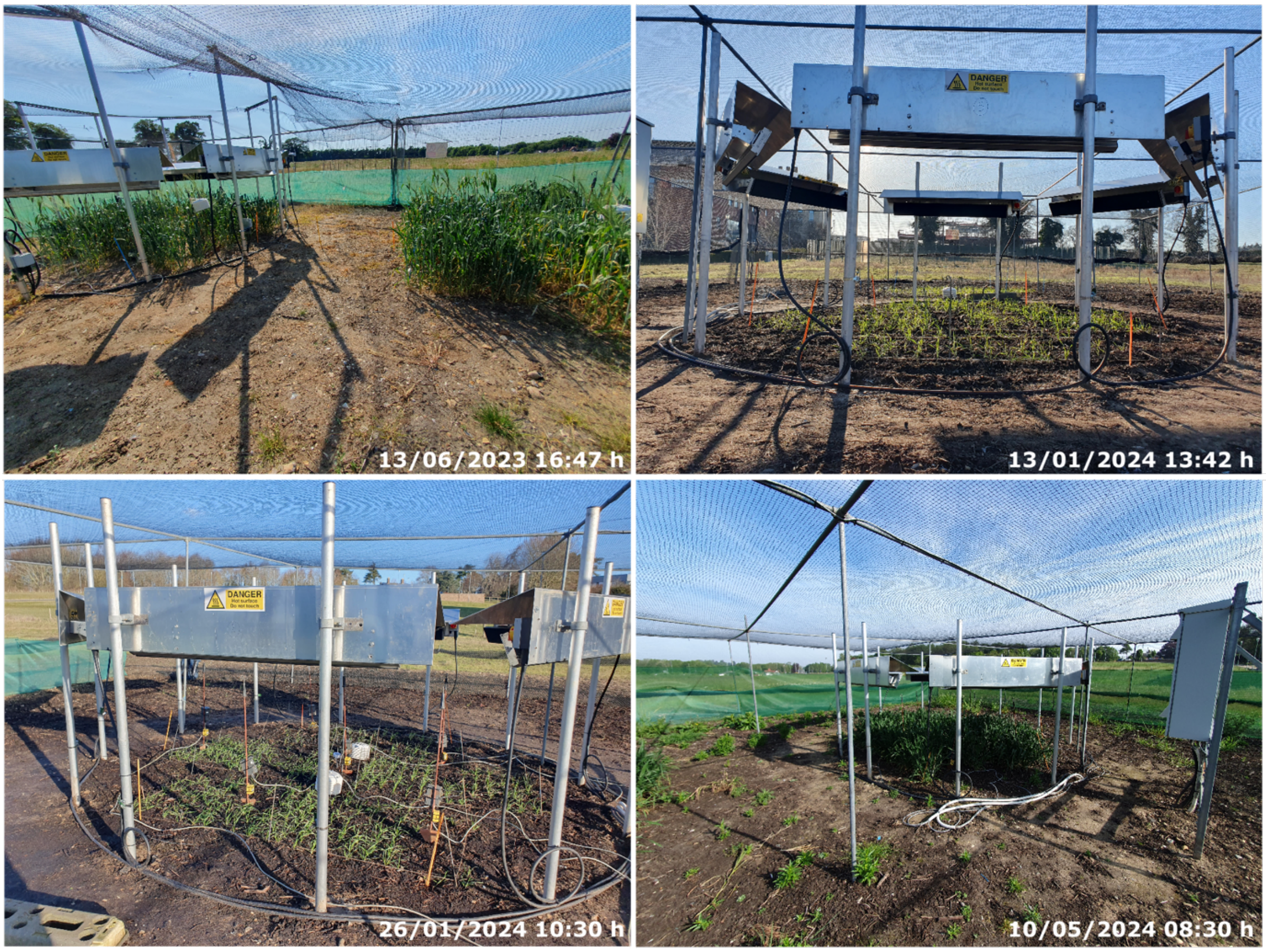

Figure S4.

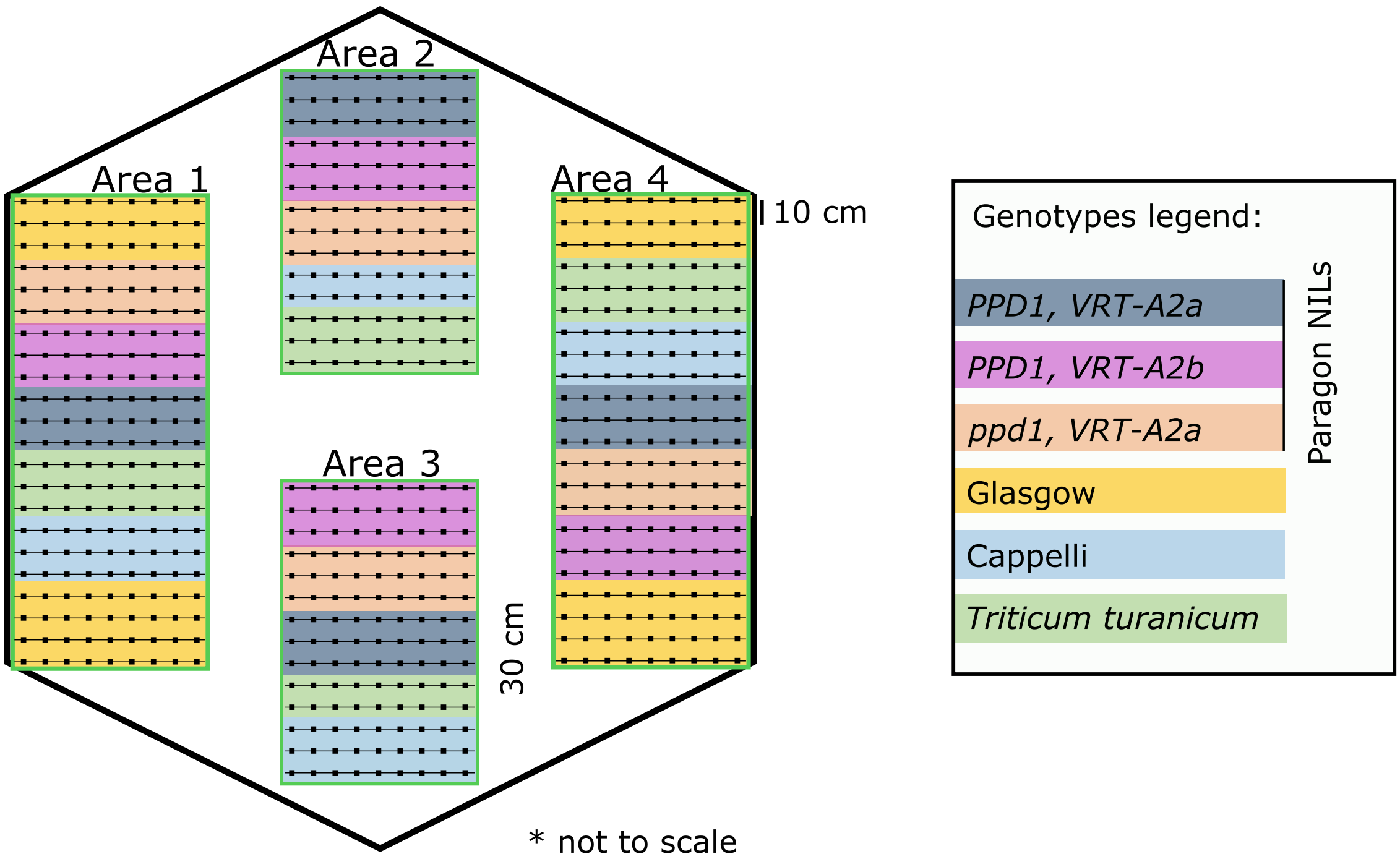

Figure S5.

Table S1.

Component	Provider	Service/components provided	Unit price	Unit quantity	Total excl. VAT	Total incl. VAT
**Scaffold**	EPS	Provide scaffold and set up scaffold, heaters and covers	NA	NA	£7,045.00	£8,454.00
**Control panel**	Pentagon	Provide and set up control panel	NA	NA	£7,613.00	£9,135.60
**Covers**	bartech-services	Provide aluminium heaters covers	NA	NA	£740.62	£888.74
**Heaters**	REXEL Ltd	IR-heaters Herschel Summit 2,600W	£329.25	7	£2,304.75	£2,765.70
**T and RH loggers**	Gemini Data Loggers Ltd	Tinytags plus 2 TGP-4500	£159.00	10	£1,590.00	£1,908.00
					**Total incl. VAT**	**£23,152.04**
