# Supplementary material for: Design and Deployment of a Regulation‐Compliant Infrared Heating System for UK Field Trials
Source: Plant Environ Interact. 2026 Sep 9;7(5):e70189. doi: 10.1002/pei3.70189 (PMC13555399; doi:10.1002/pei3.70189)
Supplement: Supplementary file 3 [file PEI3-7-e70189-s004.pdf]

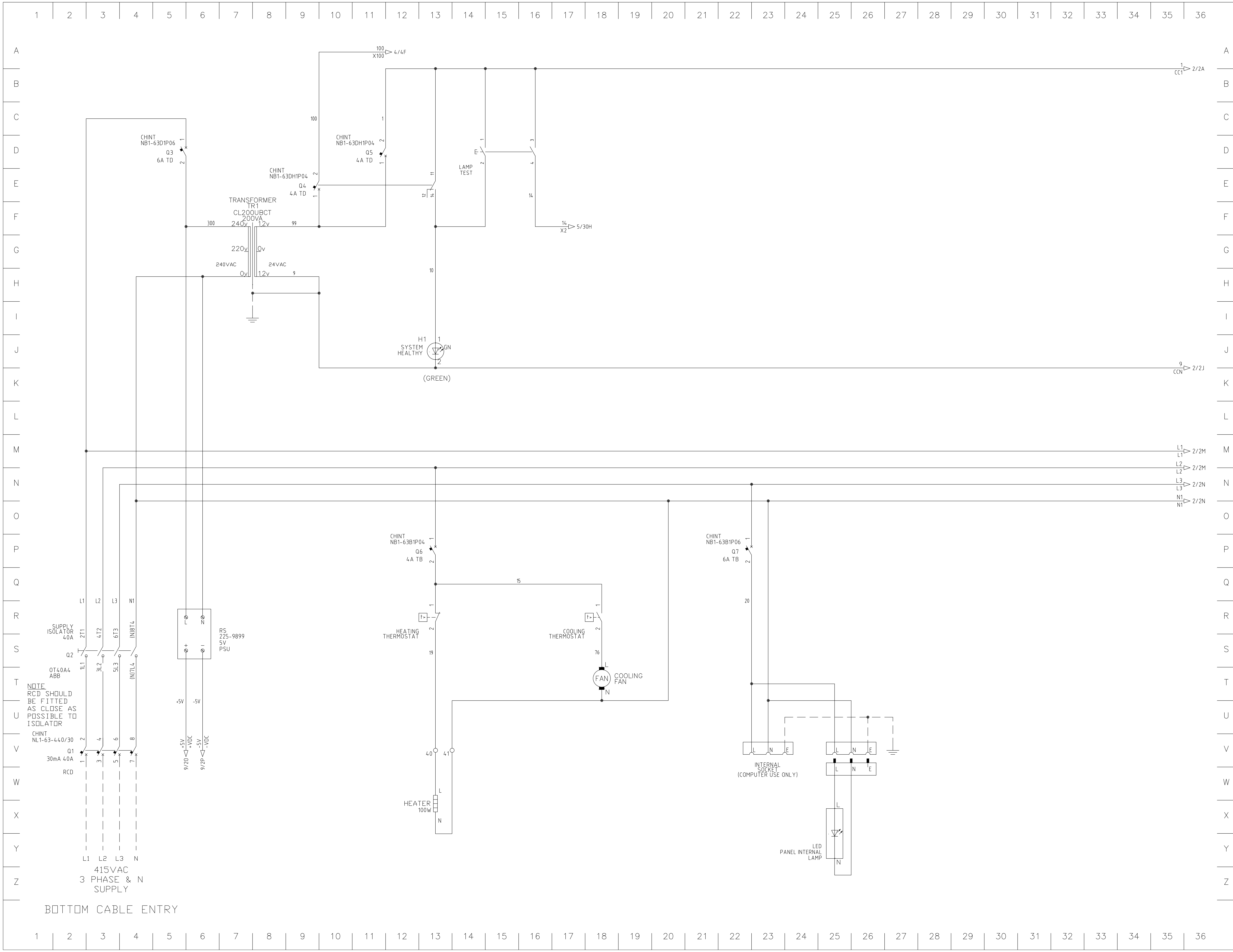

REVISION HISTORY

REV	DESCRIPTION	ENG	DATE
A	Client approval	GJC	09/11/2022
M	Manufacture	GWW	28/11/2022
T	Test	GWW	05/12/2022
T1	Heater switching changed	GJC	03/02/2023

REVISION LEGEND

A	MODIFICATIONS DURING CLIENT APPROVAL
M	MODIFICATIONS DURING MANUFACTURE
T	MODIFICATIONS DURING TESTING
AF	MODIFICATIONS DURING COMMISSIONING

DRAWING INFORMATION

CAD Ref: 1

Pentagon
Control Systems
UNIT 8
BURNET ROAD
NORWICH
NR3 2BS
TEL: (01603) 404828
FAX: (01603) 404898

PCS
HEATING & VENTILATION
CONTROL SYSTEMS

CLIENT
N.B.I.P Ltd
John Innes Centre
Norwich Research
Park, Colney
Norwich NR4 7UH

PROJECT DESCRIPTION
John Innes Centre
Field Heating Trials

DRAWING DESCRIPTION
Control Panel
Wiring Diagram

DRAWING SHEET NUMBER
1 OF 5

PROJECT NUMBER
P32700_CP001

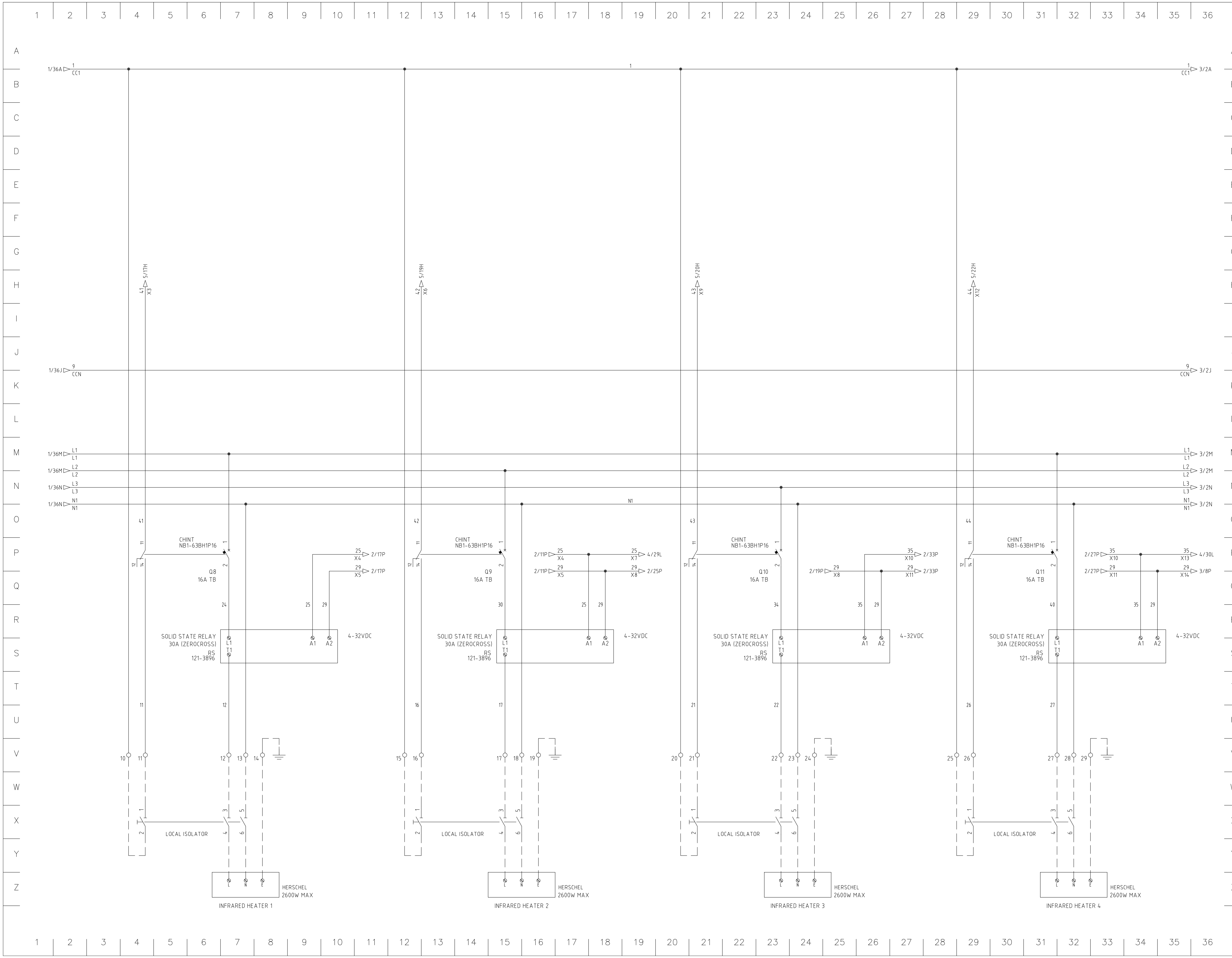

REVISION HISTORY

REV	DESCRIPTION	ENG	DATE
A	Client approval	GJC	09/11/2022
M	Manufacture	GWW	28/11/2022
T	Test	GWW	05/12/2022
T1	Heater switching changed	GJC	03/02/2023

REVISION LEGEND

A	MODIFICATIONS DURING CLIENT APPROVAL
M	MODIFICATIONS DURING MANUFACTURE
T	MODIFICATIONS DURING TESTING
AF	MODIFICATIONS DURING COMMISSIONING

DRAWING INFORMATION

CAD Ref: 2

Pentagon
Control Systems
UNIT 8
BURNET ROAD
NORWICH
NR3 2BS
TEL: (01603) 404828
FAX: (01603) 404898

PCS
HEATING & VENTILATION CONTROL SYSTEMS

CLIENT
N.B.I.P Ltd
John Innes Centre
Norwich Research
Park, Colney
Norwich NR4 7UH

PROJECT DESCRIPTION
John Innes Centre
Field Heating Trials

DRAWING DESCRIPTION
Control Panel
Wiring Diagram

PROJECT NUMBER
P32700_CP001

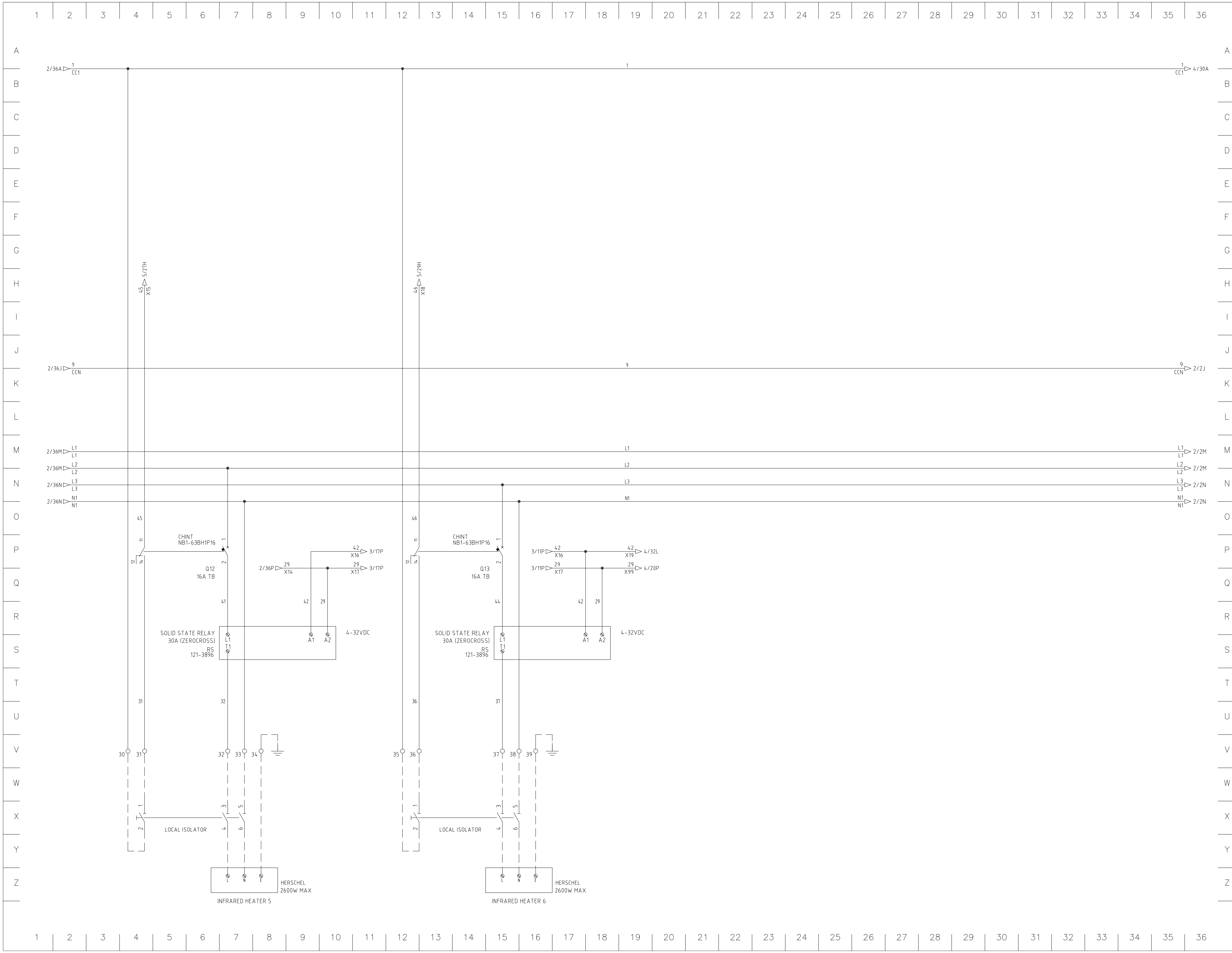

REVISION HISTORY

REV	DESCRIPTION	ENG	DATE
A	Client approval	GJC	09/11/2022
M	Manufacture	GWW	28/11/2022
T	Test	GWW	05/12/2022
T1	Heater switching changed	GJC	03/02/2023

REVISION LEGEND

A MODIFICATIONS DURING CLIENT APPROVAL
M MODIFICATIONS DURING MANUFACTURE
T MODIFICATIONS DURING TESTING
AF MODIFICATIONS DURING COMMISSIONING

DRAWING INFORMATION

CAD Ref: 3

Pentagon
Control Systems
UNIT 8
BURNET ROAD
NORWICH
NR3 2BS
TEL: (01603) 404828
FAX: (01603) 404898

HEATING & VENTILATION CONTROL SYSTEMS

CLIENT
N.B.I.P Ltd
John Innes Centre
Norwich Research
Park, Colney
Norwich NR4 7UH

PROJECT DESCRIPTION
John Innes Centre
Field Heating Trials

DRAWING DESCRIPTION
Control Panel
Wiring Diagram

DRAWING SHEET NUMBER
3 OF 5

PROJECT NUMBER
P32700_CP001

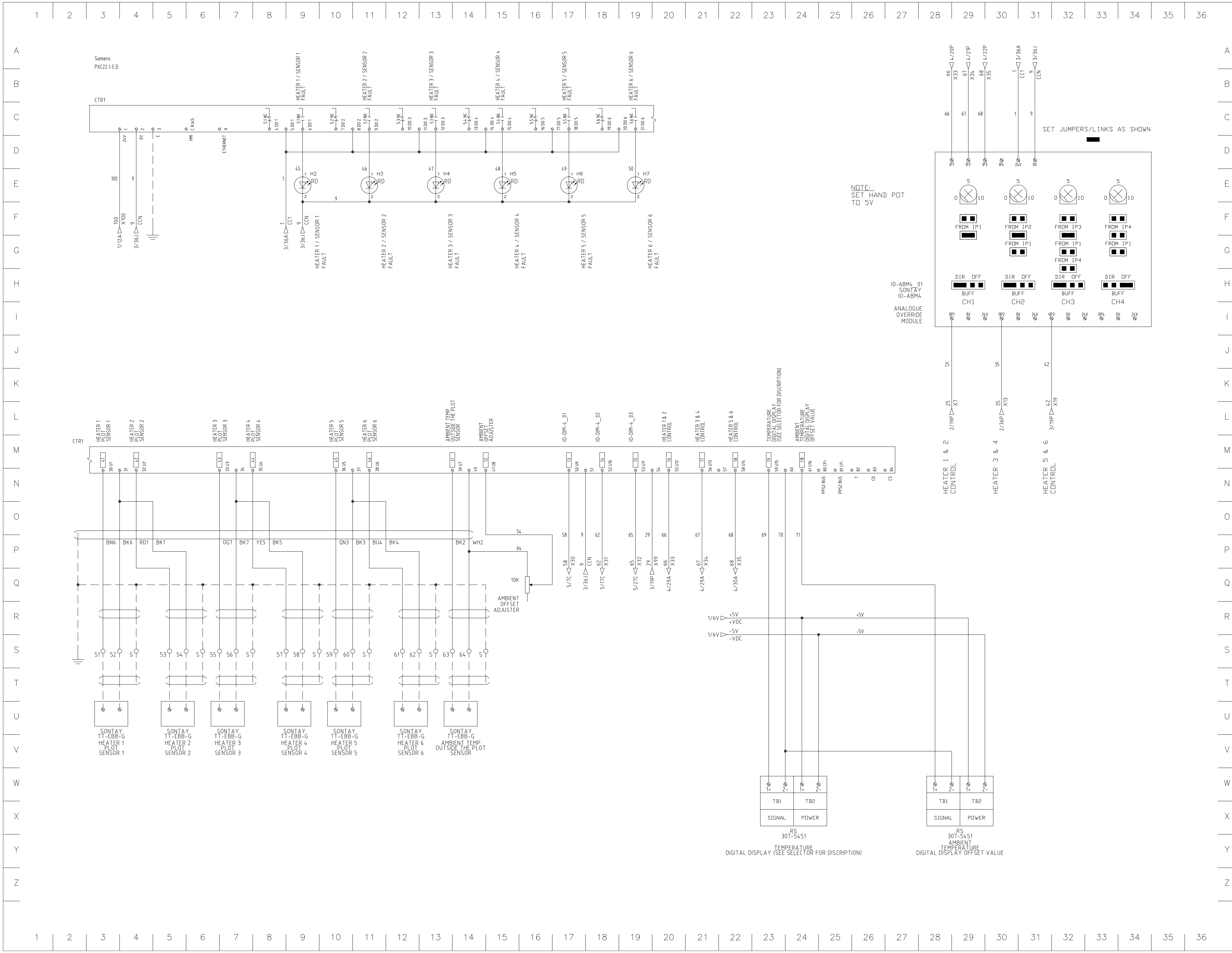

REVISION HISTORY

REV	DESCRIPTION	ENG	DATE
A	Client approval	GJC	09/11/2022
M	Manufacture	GWW	28/11/2022
T	Test	GWW	05/12/2022
T1	Heater switching changed	GJC	03/02/2023

REVISION LEGEND

A	MODIFICATIONS DURING CLIENT APPROVAL
M	MODIFICATIONS DURING MANUFACTURE
T	MODIFICATIONS DURING TESTING
AF	MODIFICATIONS DURING COMMISSIONING

DRAWING INFORMATION

CAD Ref: 4

Pentagon Control Systems
UNIT 8
BURNET ROAD
NORWICH
NR3 2BS
TEL: (01603) 404828
FAX: (01603) 404898

PCS
HEATING & VENTILATION CONTROL SYSTEMS

CLIENT
N.B.I.P Ltd
John Innes Centre
Norwich Research
Park, Colney
Norwich NR4 7UH

PROJECT DESCRIPTION
John Innes Centre
Field Heating Trials

DRAWING DESCRIPTION
Control Panel
Wiring Diagram

DRAWING SHEET NUMBER
4 OF 5

PROJECT NUMBER
P32700_CP001

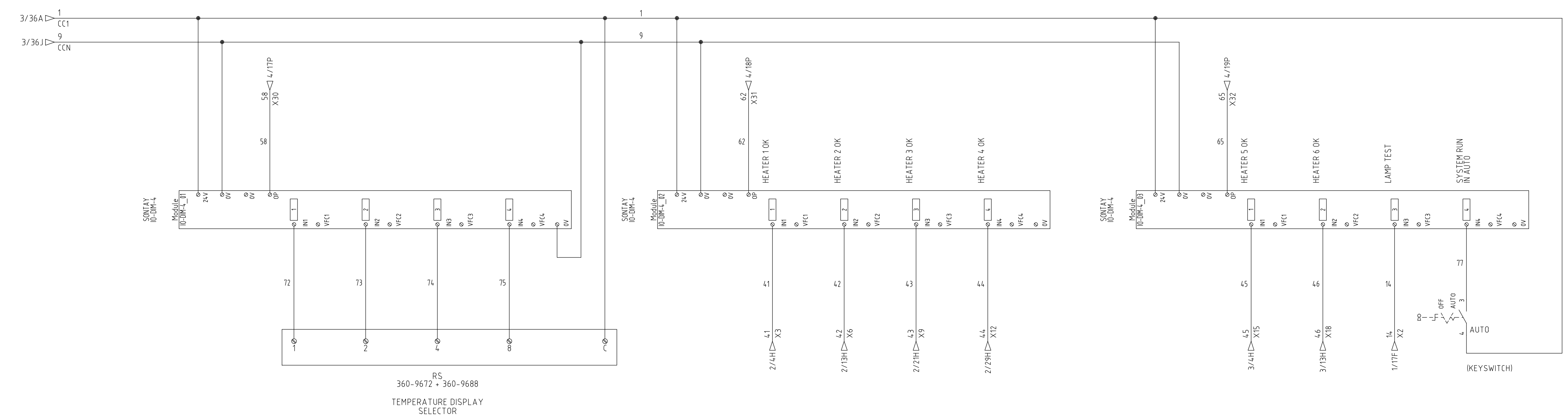

0-9 BCD PUSHWHEEL SWITCH

0-AMBIENT TEMPERATURE
1-HEATER 1 SENSOR
2-HEATER 2 SENSOR
3-HEATER 3 SENSOR
4-HEATER 4 SENSOR
5-HEATER 5 SENSOR
6-HEATER 6 SENSOR
7-MINIMUM PLOT SENSOR TEMPERATURE
8-MAXIMUM PLOT SENSOR TEMPERATURE
9-AVERAGE PLOT SENSOR TEMPERATURE

REVISION HISTORY

REV	DESCRIPTION	ENG	DATE
A	Client approval	GJC	09/11/2022
M	Manufacture	GWW	28/11/2022
T	Test	GWW	05/12/2022
T1	Heater switching changed	GJC	03/02/2023

REVISION LEGEND

A MODIFICATIONS DURING CLIENT APPROVAL
M MODIFICATIONS DURING MANUFACTURE
T MODIFICATIONS DURING TESTING
AF MODIFICATIONS DURING COMMISSIONING

DRAWING INFORMATION

CAD Ref: 5

Pentagon
Control Systems
UNIT 8
BURNET ROAD
NORWICH
NR3 2BS
TEL: (01603) 404828
FAX: (01603) 404898

HEATING & VENTILATION CONTROL SYSTEMS

CLIENT
N.B.I.P Ltd
John Innes Centre
Norwich Research
Park, Colney
Norwich NR4 7UH

PROJECT DESCRIPTION
John Innes Centre
Field Heating Trials

DRAWING DESCRIPTION
Control Panel
Wiring Diagram

DRAWING SHEET NUMBER
5 OF 5

PROJECT NUMBER
P32700_CP001
